# The inflammatory cytokine TNF-α promotes the premature senescence of rat nucleus pulposus cells via the PI3K/Akt signaling pathway

**DOI:** 10.1038/srep42938

**Published:** 2017-02-17

**Authors:** Pei Li, Yibo Gan, Yuan Xu, Lei Song, Liyuan Wang, Bin Ouyang, Chengmin Zhang, Qiang Zhou

**Affiliations:** 1 Department of Orthopedic Surgery, Southwest Hospital, Third Military Medical University, Chongqing, 400038, China; 2 Department of Orthopedic Surgery, Xinqiao Hospital, Third Military Medical University, Chongqing, 400037, China

## Abstract

Premature senescence of nucleus pulposus (NP) cells and inflammation are two common features of degenerated discs. This study investigated the effects of the inflammatory cytokine TNF-α on the premature senescence of NP cells and the molecular mechanism behind this process. Rat NP cells were cultured with or without different concentrations of TNF-α for 1 and 3 days. The inhibitor LY294002 was used to determine the role of the PI3K/Akt pathway. NP cells that were incubated with TNF-α for 3 days followed by 3 days of recovery in the control medium were used to analyze cellular senescence. Results showed that TNF-α promoted premature senescence of NP cells, as indicated by decreased cell proliferation, decreased telomerase activity, increased SA-β-gal staining, the fraction of cells arrested in the G1 phase of the cell cycle, the attenuated ability to synthesize matrix proteins and the up-regulated expression of the senescence marker p16 and p53. Moreover, a high TNF-α concentration produced greater effects than a low TNF-α concentration on day 3 of the experiment. Further analysis indicated that the inhibition of the PI3K/Akt pathway attenuated the TNF-α-induced premature senescence of NP cells. Additionally, TNF-α-induced NP cell senescence did not recover after TNF-α was withdrawn. In conclusion, TNF-α promotes the premature senescence of NP cells, and activation of the PI3K/Akt pathway is involved in this process.

Intervertebral disc degeneration (IDD) is frequently associated with low back pain (LBP), which leads to patient disability and considerable financial ruin^1^. Current treatments, including surgery and conservative therapy, are aimed at symptomatic pain alleviation rather than retarding the progression of IDD^2^. To date, the pathological mechanisms underlying this disc degeneration remain largely unclear.

During disc degeneration, the extracellular matrix within the nucleus pulposus (NP) undergoes dramatic molecular changes, such as decreased hydration, decreased proteoglycan content and alterations in collagen content^3^. These matrix changes directly reflect NP cell biology, which is indicated by the finding that NP cells display an altered gene or protein expression profile during disc degeneration degeneration^4^. Cell senescence is a cellular process that can significantly attenuate cell function^5^. Several studies report the cellular senescent phenotype within degenerated human intervertebral discs and suggest a correlation between cell senescence and disc degeneration^6^,^7^,^8^,^9^. Moreover, it has been demonstrated that the amount of senescent disc cells increases with advancing disc degeneration^9^,^10^. Therefore, we deduce that NP cell senescence may partially participate in the process of IDD.

Apart from the increase in senescent cells during disc degeneration, the accompanying inflammation within NP is also a common phenomenon during disc degeneration^11^. Many inflammatory cytokines, such as TNF-α, IL-1β and IL-17, are up-regulated in degenerated discs^12^,^13^,^14^,^15^. Previous studies demonstrated that inflammatory cytokines are often related to premature senescence of certain cell types, such as endothelial progenitor cells and osteoarthritic osteoblasts^16^,^17^,^18^. To the best of our knowledge, few studies have investigated the relationship between inflammatory cytokines and the premature senescence of NP cells.

In the present study, we investigated whether the inflammatory cytokine TNF-α induced premature senescence of rat NP cells and whether NP cells recovered from senescence after withdrawal of TNF-α. The PI3K/Akt signaling pathway plays an important role in numerous cellular activities^19^ and is also involved in the aging process of other cell types^20^,^21^. Previous data shows that the PI3K/Akt signaling pathway is activated by TNF-α^22^,^23^,^24^. Hence, the role of the PI3K/Akt signaling pathway was studied by using LY294002, a specific inhibitor that suppresses PI3K/Akt activity through inhibiting Akt phosphorylation. NP cell senescence was evaluated by measuring several senescence markers, including senescence markers (p16 and p53) expression, cell proliferation, telomerase activity, cell cycle and SA-β-Gal activity. In addition, glycosaminoglycan (GAG) content, gene expression and protein expression of matrix macromolecules (aggrecan and collagen II) were also measured to assess the matrix homeostatic phenotype of these cells.

## Materials and Methods

### Tissue harvest, cell isolation and cell culture

Thirty-five Sprague-Dawley rats (male, 250 g and 6–8 weeks old) were obtained from the Animal Center and approved by the Ethics Committee at Southwest Hospital affiliated with the Third Military Medical University. The animal care methods were carried out in accordance with the relevant guidelines [SYXK (YU) 2012–0012]. Briefly, after rats were sacrificed with excess carbon dioxide inhalation, the thoracic and lumbar discs were harvested under sterile conditions. Then, the innermost NP tissue was removed under a dissecting microscope. NP cell isolation was performed by sequential enzymatic digestion with 0.25% trypsin for 5–10 minutes and 0.25% Type I collagenase (Sigma) for 20–25 minutes at 37 °C. After digestion and centrifugation, cell pellets were re-suspended in a monolayer culture with DMEM/F12 (HyClone) containing 10% (v/v) fetal bovine serum (FBS, Gibco) and 1% (v/v) penicillin-streptomycin (Gibco) under standard conditions (37 °C, 21% O_2_ and 5% CO_2_). The culture medium was changed every 3 days and NP cells were subcultured at a ratio of 1:3 after reaching 80% confluence. To avoid the influence of cell passage, passage 2 (P2) NP cells were used in this study.

### Investigation on the direct effects of TNF-α on NP cell senescence

#### Grouping

To study the effects of TNF-α (Peprotech, recombinant human TNF-α) on NP cell senescence, P2 NP cells were assigned to the following groups: (1) a control group that was free from intervention; (2) a 10 ng/ml TNF-α group; (3) a 20 ng/ml TNF-α group; (4) a 40 ng/ml TNF-α group; and (5) a (10 ng/ml TNF-α + LY294002) group that was treated with LY294002 (10 μM, Beyotime, China) and 10 ng/ml TNF-α.

### Cell proliferation assay

NP cell proliferation was detected with a Cell Counting Kit-8 (CCK-8, Beyotime, China). Briefly, 3 × 103 cells/well were seeded in a 48-well plate and incubated with medium containing different test compounds for 1, 3 and 5 days after which they were incubated with CCK-8 solution for 2 hours at 37 °C. Then, 200 μL supernatant was used to measure the potency of cell proliferation as indicated by the absorbance at a wavelength of 450 nm. NP cell proliferation was also evaluated by the uptake of 5-ethynyl-2′-deoxyuridine (EdU) into DNA, using a Click-iT EdU microplate assay kit (Invitrogen, USA) according to the manufacturer’s instructions. Briefly, after NP cells (seeded in a 96-well plate, 1.5 × 10^3^ cells/well) were incubated with different test compounds for 1, 3 and 5 days, NP cells were labeled with EdU which was coupled to Oregon Green-azide. Then, EdU incorporated into cell DNA was detected using an HRP-conjugated anti-Oregon Green antibody and Amplex UltraRed. The NP cell proliferation rate was detected at an excitation/emission wavelength of 490/585 nm using an automatic microplate reader (Thermo, USA) and was expressed in relative fluorescence units (RFU).

### Cell viability assay

After NP cells (seeded in a 6-well plate, 5 × 10^3^ cells/well) were incubated with different test compounds for 1, 3 and 5 days, cell viability was assessed using a LIVE/DEAD Viability/Cytotoxicity Assay Kit (Invitrogen, USA). Briefly, after washing the cells 2–3 times with PBS, NP cells were incubated with a fluorescent working solution (calcein AM: 2 μM; EthD-1: 4 μM) for 40 minutes at room temperature. Then, the live (green fluorescence) or dead (red fluorescence) NP cells in each group were viewed under a fluorescence microscope (Olympus IX71, Japan). Cell viability (expressed as the live to total cell ratio) in the TNF-α-incubated groups was quantified relative to the control group (%) using Image-Pro Plus software (Version 5.1, Media Cybernetics, Inc.)

### SA-β-Gal staining

The SA-β-gal activity of cultured NP cells was measured using a Senescence β-Galactosidase Staining Kit (Beyotime, China). Briefly, NP cells were seeded in a 6-well plate (1 × 10^4^ cells/well) and grown to 50% confluence. After NP cells were incubated with medium containing different test compounds for 1 and 3 days, SA-β-Gal staining was performed according to the manufacturer’s instructions. Finally, the SA-β-Gal staining-positive NP cells were observed under a light microscope (Olympus BX51) and analyzed using Image-Pro Plus software (Version 5.1, Media Cybernetics, Inc.).

### Detection of telomerase activity

Telomerase activity of the NP cells was detected with a telomerase (TE) ELISA kit (Mlbio, China). Briefly, NP cells were seeded in a 10-cm diameter dish (1.0 × 10^4^ cells/dish) and incubated with different test compounds for 1 and 3 days. NP cell pellets were then lysed and centrifuged. Then, the supernatant was collected, and telomerase activity (IU/L) was measured according to the manufacturer’s instructions. A standard curve was developed using the standards and their respective absorbance values at 450 nm. The telomerase activity of samples was calculated based on the absorbance values at 450 nm and the standard curve.

### Cell cycle analysis

The NP cell cycle was analyzed by flow cytometry. Briefly, after an 8-hour serum starvation, NP cells were incubated with different test compounds for 1 and 3 days. Then, NP cell pellets were sequentially fixed with 75% ethanol overnight and stained with propidium iodide (PI) dye (50 μg/ml, Beyotime, China) for 30 minutes before the flow cytometry assay. The cell cycle phases (G0/G1, G2/M, S) of each group were calculated using the cell DNA content reflected by PI fluorescence intensity that was measured using the multicycle software (Japan PHENIX Company).

### Real-time PCR analysis

Expression of p53, p16, aggrecan and collagen II mRNA was analyzed by real-time PCR. Briefly, NP cells (seeded in a 6-well plate, 1 × 10^4^ cells/well) were incubated with medium containing different test compounds for 1 and 3 days. Total RNA was extracted using TRIzol reagent (Invitrogen). After the complementary DNA was synthesized using a reverse transcription kit (Roche), real-time PCR reaction was carried out. The thermal cycling for all reactions was as follows: 5 min at 95 °C, followed by 40 amplification cycles of 30 seconds at 95 °C, 30 seconds at 56 °C and 30 seconds at 72 °C. Primers of target genes (Supplementary Table S1) were synthesized by Sangon, Biotech Co., Ltd., China. GAPDH was used as the reference gene and the relative gene expression was calculated as 2^−ΔΔCt^.

### GAG content measurement

To quantify the GAG content synthesized by the NP cells, a DMMB assay was performed according to a previous method^25^. Briefly, after NP cells (seeded in a 6-well plate, 1 × 10^4^ cells/well) were incubated with different test compounds for 1 and 3 days, NP cells were collected and suspended in PBS containing 5 mg/mL papain (Sangon, Biotech Co., Ltd., China) for 6–8 hours at 60 °C. Then, the GAG content in the digested sample was measured using the 1,9-dimethyl methylene blue (DMMB) assay, in which shark cartilage chondroitin sulfate (Sigma, USA) was used as a standard, and the absorbance at 525 nm was measured.

### Immunocytochemistry analysis

Immunostaining was performed to observe production of matrix macromolecules (aggrecan and collagen II). Briefly, after incubation with medium containing different test compounds for 1 and 3 days, NP cells were fixed with 4% paraformaldehyde, permeabilized with 0.1% Triton X-100 and blocked with 5% bovine serum albumin. Then, incubation with primary antibodies (Supplementary Table S2) at 4 °C overnight and incubation with corresponding peroxidase-conjugated secondary antibodies (Supplementary Table S2) at 37 °C for 2 hours were sequentially performed. After color development with diaminobenzidine (DAB), immunostaining was observed under light microscope (Olympus BX51) and analyzed using Image-Pro Plus software (Version 5.1, Media Cybernetics, Inc.).

### Western blotting assay

NP cells were seeded in a 6-well plate and grown to 70–80% confluence. After incubation with medium containing different test compounds for 1 and 3 days, total protein was extracted using RIPA solution (Beyotime, China) and protein concentration was measured with a BCA kit (Beyotime, China). Then, protein samples were subjected to SDS-PAGE as previously described^22^. The primary and secondary antibodies used in this assay are listed in Supplementary Table S2. Protein bands on the PVDF membrane were visualized using the SuperSignal West Pico Trial Kit (Thermo). Protein expression was analyzed using Image J software (National Institutes of Health, USA) and normalized to β-actin.

### Investigation on NP cell senescence after withdrawing TNF-α

To study whether NP cell senescence can recover by withdrawing the inflammatory cytokine TNF-α, NP cells were assigned into the following groups: (1) control group that was free from intervention; (2) TNF-α withdrawal group that involved 3 days of TNF-α (10 ng/ml) exposure followed by 3 days of recovery in the control culture medium (3) TNF-α group that involved 6 days of TNF-α (10 ng/ml) exposure. After completion of the culture period, NP cells were analyzed for SA-β-Gal activity, cell cycle and the protein expression of the senescence markers p16 and p53 as described above.

### Statistics

Data were expressed as the mean ± SD and SPSS 13.0 software was used to analyze the data for statistical difference. When the homogeneity test for variance was completed, comparisons between two groups were performed using the Independent-Samples T Test. Comparisons between multiple groups with different concentrations of TNF-α (10, 20 and 40 ng/mL) was analyzed using a one-way analysis of variance (ANOVA), and the post hoc test was determined by the LSD test. A statistically significant difference was indicated when the p-value < 0.05.

## Results

### Investigation of the direct effects of TNF-α on NP cell senescence

#### TNF-α significantly attenuated the proliferation of NP cells

**Figure 1:**
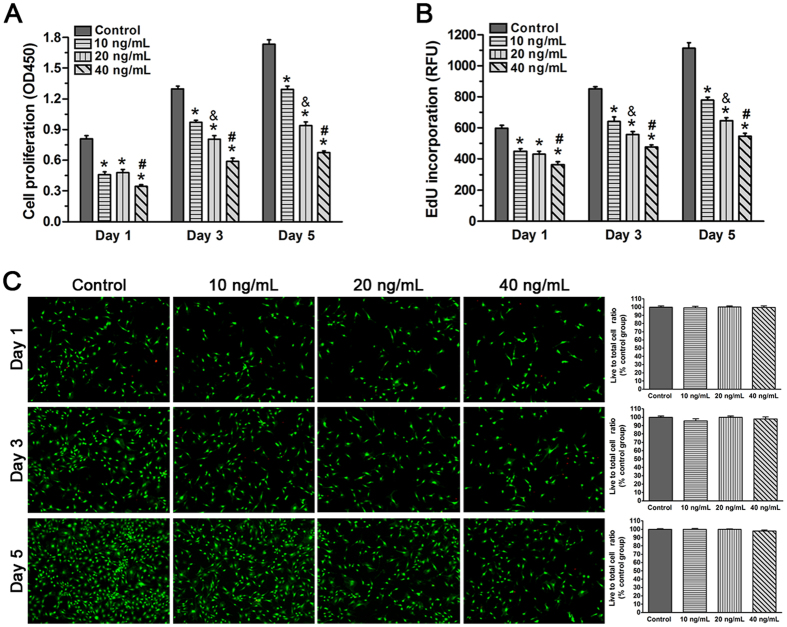
Effects of TNF-α on proliferation and viability of rat nucleus pulposus (NP) cells on day 1, 3 and 5. (**A** and **B**) CCK-8 assay and EdU incorporation assay showed that NP cell proliferation decreased as the TNF-α concentration increased. (**C**) No significant difference in NP cell viability was found between the different TNF-α concentration groups. The data are expressed as the mean ± SD (n = 3). *p < 0.05, vs. the control group; ^#^p < 0.05, vs. the group with 10 ng/mL TNF-α and group with 20 ng/ml TNF-α; ^&^p < 0.05, vs. the group with 10 ng/mL TNF-α.

Senescent cells often have limited ability of cell proliferation. To investigate how TNF-α treatment affects NP cell proliferation, we incubated NP cells with different TNF-α concentrations and analyzed cell proliferation on days 1, 3 and 5. After incubation with medium containing either 10, 20 or 40 ng/mL of TNF-α for 1, 3 and 5 days, the CCK-8 assay and EdU incorporation assay showed that NP cell proliferation was significantly inhibited compared with the control group (Fig. 1A,B). Moreover, the high concentration of TNF-α attenuated cell proliferation of the NP cell compared with the low concentration of TNF-α on days 3 and 5. However, the range of 0–40 ng/mL TNF-α did not significantly affect NP cell viability on days 1, 3 and 5 (Fig. 1C). Together, these results indicate that TNF-α inhibits NP cell proliferation in a dose-dependent manner.

#### TNF-α increased SA-β-Gal activity, reduced telomerase activity and promoted G1 cell cycle arrest of NP cells

**Figure 2:**
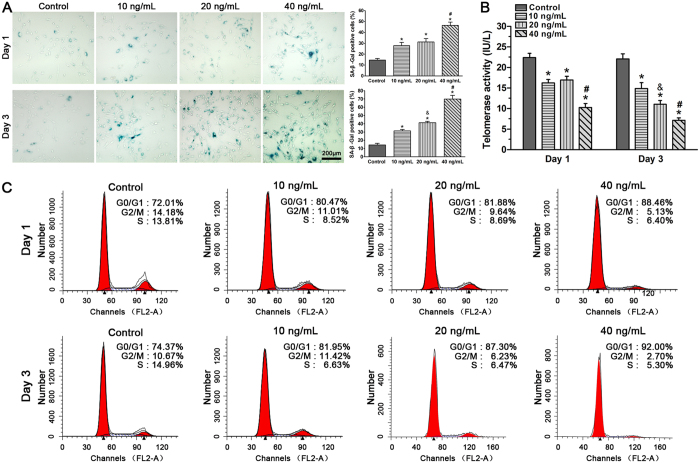
Effects of TNF-α on SA-β-Gal activity (**A**), telomerase activity (**B**) and G1 cell cycle arrest (**C**) in rat nucleus pulposus (NP) cells on day 1 and day 3. Results showed that TNF-α significantly increased SA-β-Gal activity, decreased telomerase activity and aggravated G1 cell cycle arrest of NP cells in a dose-dependent manner on day 3. The data are expressed as the mean ± SD (n = 3). *p < 0.05, vs. the control group; ^#^p < 0.05, vs. the group with 10 ng/mL TNF-α and group with 20 ng/ml TNF-α; ^&^p < 0.05, vs. the group with 10 ng/mL TNF-α.

Increased SA-β-Gal activity, decreased telomerase activity and G1 cell cycle arrest are classic characteristics of senescent cells^26^,^27^,^28^. In this study, a decreased SA-β-Gal activity was measured for NP cells treated with 10 ng/ml (27.9% positive on day 1 and 31.3% positive on day 3) and 20 ng/ml (31.2% positive on day 1 and 41.2% positive on day 3) compared with 40 ng/ml TNF-α (46.5% positive on day 1 and 70.0% positive on day 3) while all TNF-α-treated NP cells showed an increase compared to the control NP cells (14.3% positive on day 1 and 14.5% positive on day 3). Although no significant differences between groups treated with 10 and 20 ng/mL TNF-α were found on day 1, a dose-dependent response in SA-β-galactosidase activity to TNF-α appeared on day 3 (Fig. 2A).

Similarly, telomerase activity of the NP cells was decreased compared with that of the control NP cells after incubation with TNF-α on day 1 and day 3. Though no significant difference was found between the 10 and 20 ng/mL of TNF-α on day 1, an obvious dose response of telomerase activity to TNF-α was found on day 3 (Fig. 2B).

We also used the PI staining method to analyze the G1 phase fraction of NP cells after the TNF-α incubation. Results showed that a decreased G1 phase fraction was measured for NP cells treated with 10 ng/ml (80.47% positive on day 1 and 81.95% positive on day 3) and 20 ng/ml (81.88% positive on day 1 and 87.30% positive on day 3) compared with 40 ng/ml TNFa (88.46% positive on day 1 and 92.0% positive on day 3). All NP cells treated with TNF-α showed an increase in the number of cells arrested in the G1 phase of the cell cycle compared with the control NP cells (72.01% positive on day 1 and 74.37% positive on day 3) (Fig. 2C). Collectively, these findings suggest that TNF-α can accelerate NP cell senescence, as indicated by increased the SA-β-Gal activity, reduced telomerase activity and the increased number of NP cells arrested in the G1 phase of the cell cycle.

### TNF-α inhibited the synthesis of the extracellular matrix in NP cells

**Figure 3:**
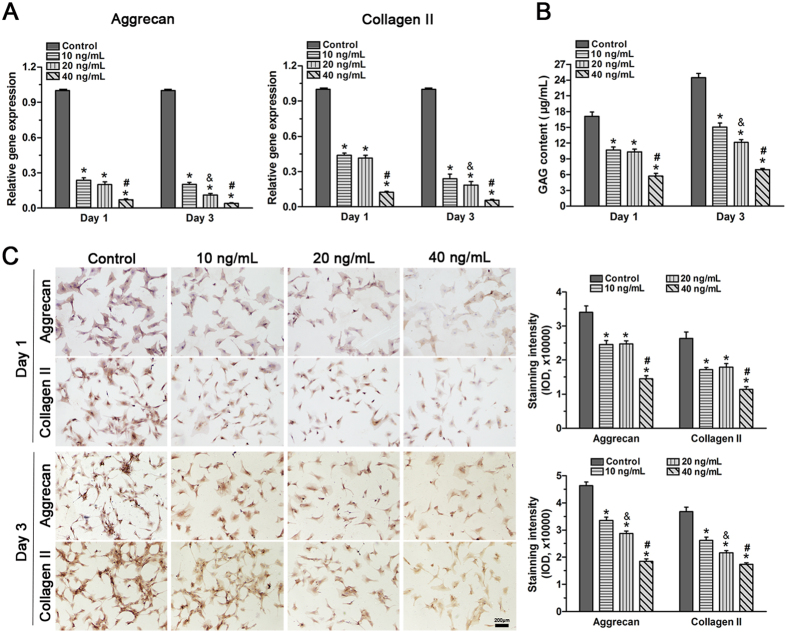
Effects of TNF-α on the gene expression of matrix molecules (**A**), glycosaminoglycan (GAG) content (**B**) and matrix deposition (**C**) in rat nucleus pulposus (NP) cells on day 1 and day 3. Results showed that TNF-α significantly down-regulated the gene expression of matrix molecules, decreased GAG content and the matrix deposition of NP cells in a dose-dependent manner on day 3. The data are expressed as the mean ± SD (n = 3). *p < 0.05, vs. the control group; ^#^p < 0.05, vs. the group with 10 ng/mL TNF-α and group with 20 ng/ml TNF-α; ^&^p < 0.05, vs. the group with 10 ng/mL TNF-α.

Homeostatic matrix biosynthesis in NP cells is important for normal disc function^29^. Senescent cells have been proposed to have altered gene and protein expression levels that may be detrimental to a healthy disc NP matrix^10^,^30^. We evaluated the expression of macromolecules and the proteoglycan production of NP cells treated with TNF-α. Real-time PCR indicated that aggrecan and collagen II gene expression in NP cells was significantly decreased after incubation with TNF-α on day 1 and day 3 (Fig. 3A). Similarly, the DMMB assay demonstrated that GAG content was reduced by TNF-α incubation, and that on day 3 this occurred in a dose-dependent manner (Fig. 3B). Additionally, immunocytochemistry showed that TNF-α significantly decreased protein deposition of these matrix macromolecules and that an obvious dose response of matrix protein deposition to TNF-α concentration appeared on day 3 (Fig. 3C). These results indicate that TNF-α promoted the development of a catabolic phenotype of matrix homeostasis in NP cells.

### Inflammatory cytokine TNF-α up-regulated expression of senescence markers in NP cells

**Figure 4:**
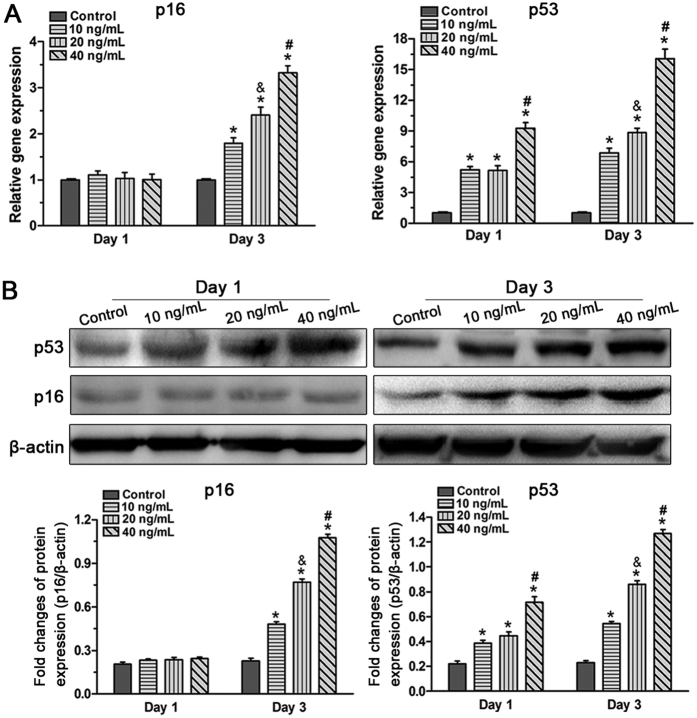
Effects of TNF-α on gene (**A**) and protein (**B**) expression of senescence markers (p16 and p53) in rat nucleus pulposus (NP) cells on day 1 and day 3. Real-time PCR analysis and western blot assay showed that TNF-α up-regulated the gene and protein expression of p16 and p53. The data are expressed as the mean ± SD (n = 3). *p < 0.05, vs. the control group; ^#^p < 0.05, vs. the group with 10 ng/mL TNF-α and group with 20 ng/ml TNF-α; ^&^p < 0.05, vs. the group with 10 ng/mL TNF-α.

Because the p53-p21 pathway and the p21-pRb pathway are closely related with cellular senescence^31^, we evaluated the effects of TNF-α on the expression of p53 and p16 both at the gene (Fig. 4A) and protein (Fig. 4B) levels. Results showed that TNF-α had no obvious effects on the expression of p16 but significantly up-regulated the expression of p53 on day 1. However, both p16 and p53 were significantly up-regulated on day 3 compared with the control group of NP cells. Moreover, NP cells treated with high TNF-α produced higher p16 and p53 levels than those treated with low TNF-α concentration. These results indicate a dose-dependent effect of TNF-α on the expression of these senescence markers.

### The PI3K/Akt signaling pathway was activated by TNF-α in NP cells

**Figure 5:**
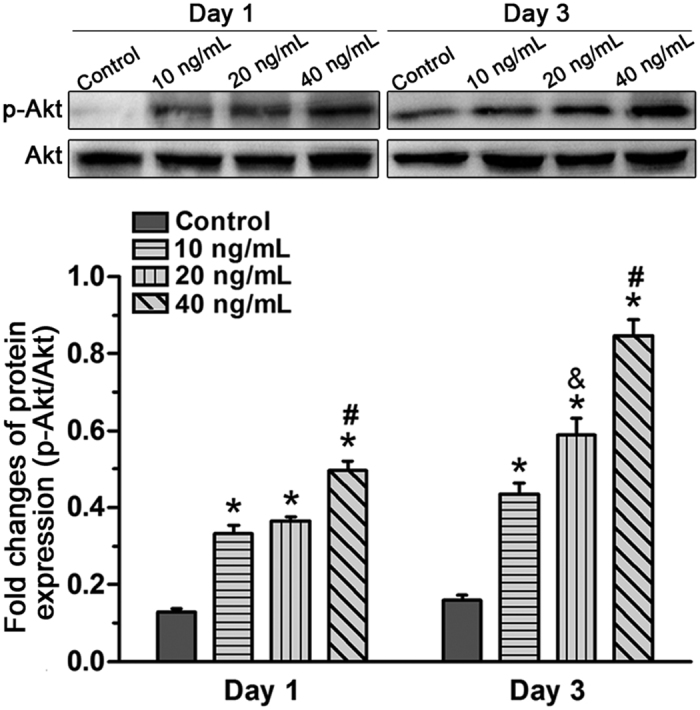
Effects of TNF-α on the activation of the PI3K/Akt signaling pathway in rat nucleus pulposus (NP) cells on day 1 and day 3. A western blot assay indicated that p-Akt expression increased after TNF-α treatment which was dose-dependent on day 3. The data are expressed as the mean ± SD (n = 3). *p < 0.05, vs. the control group; ^#^p < 0.05, vs. the group with 10 ng/mL TNF-α and group with 20 ng/ml TNF-α; ^&^p < 0.05, vs. the group with 10 ng/mL TNF-α.

The PI3K/Akt signaling pathway plays an important role in numerous cellular activities including cell senescence^19^,^20^,^21^. To investigate the effects of TNF-α on the activity of the PI3K/Akt pathway, the protein expression of p-Akt was evaluated by western blot. Results showed that the expression of p-Akt significantly increased with TNF-α on day 1 and day 3. In addition, though no significant difference was found between the 10 ng/ml TNF-α and 20 ng/ml TNF-α treatment on day 1, an increased expression of p-Akt was found in NP cells treated with a high TNF-α concentration compared with NP cells treated with a low TNF-α concentration on day 3 (Fig. 5). These findings suggest that the PI3K/Akt signaling pathway was activated by TNF-α in NP cells.

### Inhibition of the PI3K/Akt signaling pathway attenuated TNF-α-induced premature senescence of NP cells

**Figure 6:**
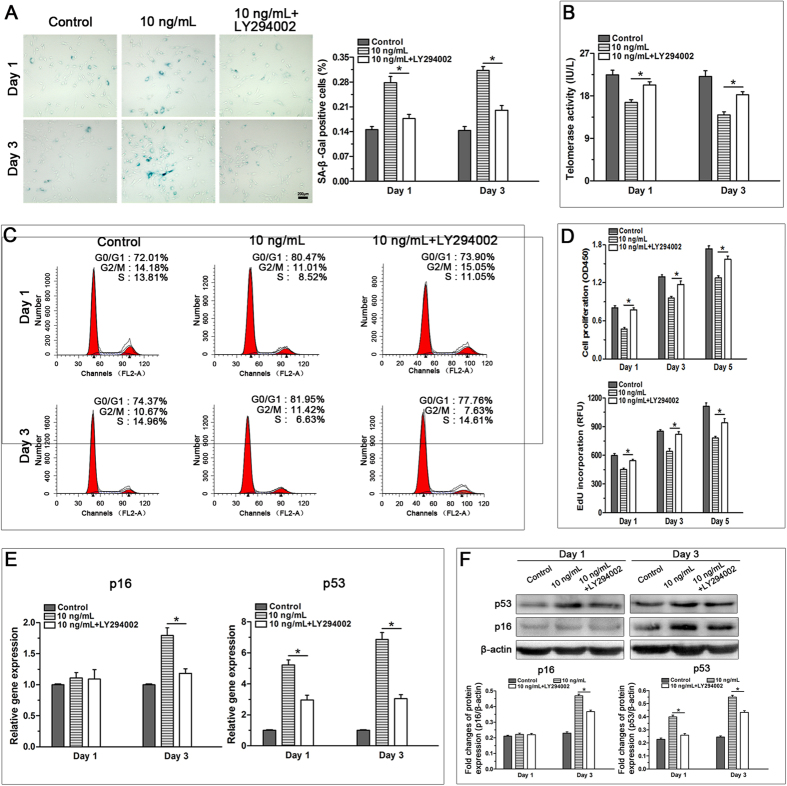
Inhibition of the PI3K/Akt signaling pathway attenuated the TNF-α-induced senescence phenotype of rat nucleus pulposus (NP) cells on day 1 and day 3. Results showed that LY294002 decreased SA-β-Gal activity (**A**), increased telomerase activity (**B**), attenuated G1 cell cycle arrest (**C**), promoted cell proliferation (**D**) and down-regulated gene and protein expression of p16 and p53 (**E** and **F**) in TNF-α-treated NP cells. The data are expressed as the mean ± SD (n = 3). *Indicates a significant difference (p < 0.05) between the 10 ng/mL TNF-α group and the 10 ng/mL TNF-α + LY294002 group.

To investigate the role of the PI3K/Akt signaling pathway on the regulatory effects of TNF-α on the premature senescence of NP cells, we treated NP cells with LY294002 and 10 ng/mL TNF-α, and verified efficiency of LY294002 in inhibiting the activation of PI3K/Akt signaling pathway (Supplementary Figure S1A). Results showed that LY294002 significantly decreased SA-β-Gal activity (Fig. 6A), restored telomerase activity (Fig. 6B) and abrogated G1 cell cycle arrest of TNF-α-treated NP cells (Fig. 6C). Furthermore, PI3K/Akt inhibition significantly promoted the proliferation potential of NP cells treated with TNF-α (Fig. 6D), whereas there was no significant difference in cell viability before and after inhibiting the activation of the PI3K/Akt pathway (Supplementary Figure S1B). Additionally, LY294002 down-regulated the expression of senescence markers (p16 and p53) (Fig. 6E,F) and promoted matrix biosynthesis (Supplementary Figure S1C–E) in NP cells treated with TNF-α. Together, these findings indicate that the premature senescence of NP cells caused by TNF-α is mediated partly by the PI3K/Akt signaling pathway.

### Effect of TNF-α withdrawal on NP cell senescence

**Figure 7:**
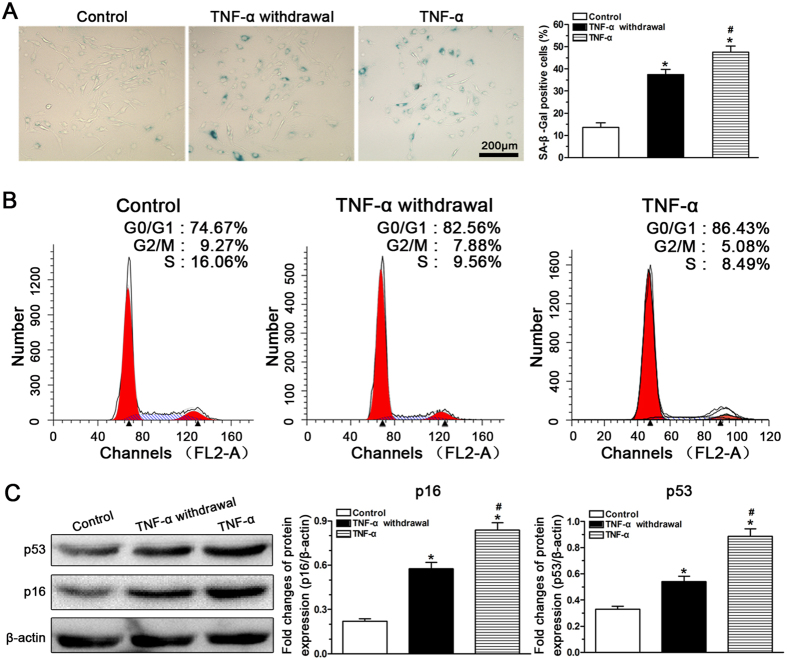
Investigation of rat nucleus pulposus (NP) cell senescence after TNF-α withdrawal. When TNF-α was withdrawn, SA-β-Gal activity (**A**), G1 cell cycle arrest (**B**) and the expression of senescence markers (**C**) in the TNF-α withdrawal group were still increased compared with those in the control group. The data are expressed as the mean ± SD (n = 3). *Indicates a significant difference (p < 0.05) compared with the control group. ^#^Indicates a significant difference (p < 0.05) between the TNF-α group and the TNF-α withdrawal group.

To investigate whether TNF-α-induced NP cell senescence was reversible once TNF-α was withdrawn, a comparison of the NP cell senescence-associated phenotype among the control, TNF-α-treated and TNF-α-withdrawal groups was performed. Results showed that the number of SA-β-Gal staining positive NP cells in the TNF-α withdrawal group (37.5%) significantly increased compared with that in the control group (13.6%) and decreased compared with that in the TNF-α group (47.6%) (Fig. 7A). Consistently, an increased G1 phase fraction was measured for NP cell in the TNF-α group (86.43%) compared with that in the TNF-α withdrawal group (82.56%) and the control group (74.67%) (Fig. 7B). Additionally, protein expression of the senescence markers p16 and p53 was significantly up-regulated in the TNF-α withdrawal group and TNF-α group compared with the control group. The TNF-α group had higher p16 and p53 expression than the TNF-α withdrawal group (Fig. 7C). Taken together, these results indicate that TNF-α-induced NP cell senescence cannot be recovered by withdrawing TNF-α.

## Discussion

The incidence of disc degeneration increases with aging. In recent years, cellular senescence is increasingly reported in degenerate disc and is suggested to be positively related with disc degeneration^6^,^9^,^10^,^32^. Inflammatory within the NP region is another important pathological phenomenon during disc degeneration. To the best of our knowledge, this is the first study to specifically explore the effects of the inflammatory cytokine TNF-α on the premature senescence of NP cells and the potential mechanism behind this regulatory process. TNF-α significantly promoted premature senescence of NP cells *in vitro*, as shown by our data on cell proliferation, SA-β-Gal staining, telomerase activity, cell cycle, and expression of matrix macromolecules and senescence markers. The inhibition of the PI3K/Akt signaling pathway attenuated TNF-α-induced NP cell senescence. Moreover, TNF-α-induced NP cell senescence was not recoverable after the withdrawal of TNF-α. These findings provide a theoretical basis for the relationship between inflammatory cytokines and NP cell senescence and will ultimately improve our understanding of disc degeneration.

SA-β-galactosidase activity is a useful marker for senescent cells^26^. In this study, TNF-α significantly increased the number of NP cells positively stained for SA-β-Gal. In addition, alternative indirect parameters, including cell proliferation and telomerase activity were also used to evaluate NP cell senescence. Results showed that the proliferation potential and telomerase activity of NP cells were attenuated by TNF-α. These findings indicate that TNF-α has an important role in promoting the premature senescence and growth arrest of NP cells. Senescent cells are often arrested in the G1 phase of the cell cycle^28^. Our data showed that TNF-α-treated NP cells were arrested in the G1 phase of cell cycle compared with the control NP cells, suggesting again that NP cell growth arrest can be exacerbated by TNF-α. A previous study indicated that, with increasing culture passages, NP cells exhibited features of senescence^33^. Similarly, the fraction of normal NP cells in the G1 phase is estimated to be approximately 70% according to a previous study^34^ and our own experience. Hence, we deduce the relative high percentage of G1 phase fraction for the control NP cells in this study is largely due to natural senescence and increased cell passage number.

The current opinion on cell senescence proposes that although senescent cells commonly become insensitive to mitogenic stimuli, their altered gene and protein expression activities may affect matrix metabolism^10^,^30^. In this study, we found that NP cells incubated with TNF-α presented decreased gene and protein expression of the NP matrix macromolecules aggrecan and collagen II compared with the control cells. This indicates that the matrix synthesis ability in TNF-α-treated NP cells is attenuated. These data agree with those of Dimozi *et al*.^35^ who reported that hydrogen peroxide-induced senescent NP cells possessed a catabolic phenotype characterized by increased matrix degradation enzymes and decreased tissue inhibitors of metalloproteinases and proteoglycans. Hence, acceleration of NP cell senescence caused by any adverse stimuli may partially contribute to the impairment of NP repair during disc degeneration and/or disc aging. A previous study by Le *et al*.^9^ indirectly supports this, showing a positive correlation between cellular senescence and the expression of the matrix catabolism-related enzymes MMP-13 and ADAMTS-5.

Two mechanisms are responsible for the induction of cellular senescence: the p53-p21-pRB pathway represents replicative senescence (RS), and the p16-pRB pathway represents stress-induced premature senescence (SIPS)^36^. In this study, we found that TNF-α significantly up-regulated the expression of p53 and p16 both at the gene and protein levels, though the expression of p16 on day 1 remained unchanged. This finding suggests that both RS and SIPS play an important role in TNF-α-induced NP cell senescence and that activation of the stress-based SIPS in NP cells may be related to TNF-α exposure time. Physiologically, NP cells are subjected to numerous insults including mechanical load, oxidative stress and increased inflammatory cytokines^9^,^37^,^38^. Therefore, the p53-p21-pRB pathway and the p16-pRB pathway may both participate in NP cell senescence in complex *in vivo* situations. Consistent with this, a previous study demonstrated that p16 and p53, though uncommon, are co-expressed in clinical disc samples from patients^36^, further supporting the hypothesis that there is a situation in which the two senescent pathways are activated simultaneously.

In our study, TNF-α significantly increased the expression of phospho-Akt in a dose-dependent manner. However, when Akt phosphorylation was inhibited by inhibitor LY294002, NP cell senescence was abrogated, as reflected by decreased SA-β-galactosidase activity, decreased p16 and p53 expression, increased cell proliferation increased matrix macromolecule expression, and attenuated G1 cell cycle arrest. These findings indicate that the PI3K/Akt pathway is involved in the effects of TNF-α on NP cell senescence. Consistent with this, a previous study demonstrated that the PI3K/Akt signaling pathway participates in inflammation-induced mesenchymal stem cell senescence^20^. Nevertheless, several other signaling pathways, including the p38-MAPK and nuclear factor-κB (NF-κB) pathways, can be activated by inflammatory cytokines^39^,^40^. Recently increasing evidence demonstrates the significance of these two signaling pathways in the pathogenesis of disc disease^41^. Moreover, both the p38-MAPK pathway and the NF-κB pathway are upstream signals of cell senescence^16^,^42^,^43^. Akt also induces cell senescence by regulating p53 in the cell senescence pathway^44^,^45^. There may be crosstalk between the PI3K/Akt pathway and the p38-MAPK pathway or the NF-κB pathway in inflammatory cytokine-induced NP cell senescence. However, the accurate molecular mechanisms of inflammatory cytokine-induced NP cell senescence need to be studied further.

Because previous studies indicated that NP cell apoptosis could be induced by high doses of TNF-α (≥50 ng/mL, usually at least 100 ng/mL)^46^,^47^,^48^,^49^, a conservative range of TNF-α concentration (0–40 ng/mL) was chosen in this study. This range of TNF-α concentrations is higher than the usually used TNF-α concentrations (0.1–10 ng/mL) in other cell types, possibly because cytokines, such as TNF-α, are known to interact and bind proteoglycans which can be released into the culture medium by NP cells^50^, thereby decreasing the effective concentration that NP cells experience. Because 10 ng/mL TNF-α induced NP cell senescence and a significant activation of the PI3K/Akt signaling pathway, only 10 ng/mL TNF-α was used to investigate the role of PI3K/Akt signaling pathway. According to our results, it is possible that the inhibitor LY294002 may also inhibit the senescent phenotype of NP cells treated with 20 or 40 ng/mL TNF-α. It is also well known that cellular senescence is typically irreversible. To investigate whether the senescent NP cells could recover after TNF-α was withdrawn, we designed an experiment where cells were exposed to 10 ng/mL TNF-α followed by incubation with the control medium, and we showed that TNF-α-induced NP cell senescence could not recover after TNF-α withdrawal.

This study also has some limitations. In this study, NP cells were plate-cultured under normoxic conditions that differ from the physiological conditions in which NP cells are contained in a three-dimensional (3D) environment under hypoxic conditions. However, this plate-culture system showed an obvious effect of TNF-α on NP cell senescence. So this study may be convincing to some extent. In fact, we had developed a 3D hydrogel system that consists of glucan, gelatin and polyethylene glycol, which we will use to study the effects of TNF-α on NP cell senescence in the future.

In conclusion, our data demonstrate that TNF-α promoted NP cell senescence *in vitro* and that the PI3K/Akt signaling pathway is involved in this regulatory process. This study sheds light on the relationship between inflammatory cytokines and NP cell senescence.

## Additional Information

**How to cite this article:** Li, P. *et al*. The inflammatory cytokine TNF-α promotes the premature senescence of rat nucleus pulposus cells via the PI3K/Akt signaling pathway. *Sci. Rep.* **7**, 42938; doi: 10.1038/srep42938 (2017).

**Publisher's note:** Springer Nature remains neutral with regard to jurisdictional claims in published maps and institutional affiliations.

## Supplementary Material

Supplementary Figures and Tables
