# Supplementary material for: The inflammatory cytokine TNF-α promotes the premature senescence of rat nucleus pulposus cells via the PI3K/Akt signaling pathway
Source: Sci Rep. 2017 Feb 17;7:42938. doi: 10.1038/srep42938 (PMC5314336; doi:10.1038/srep42938)

Inflammatory cytokine TNF-α promotes premature senescence of rat nucleus pulposus cells through PI3K/Akt signaling pathway

Pei Li [1], Yibo Gan [1], Yuan Xu [2], Lei Song [1], Liyuan Wang [1], Bin Ouyang [1], Chengmin Zhang [1], Qiang Zhou [1,*]

Supplementary Table S1. Primers of target genes

Gene	Accession number	Forward (5'-3')	Reverse (5'-3')
β-actin	NM_031144.3	CCGCGAGTACAACCTTCTTG	TGACCCATACCCACCATCAC
Aggrecan	XM_002723376.1	ATGGCATTGAGGACAGCGAA	GCTCGGTCAAAGTCCAGTGT
Collagen II	NM_012929.1	GCCAGGATGCCCGAAAATTAG	CCAGCCTTCTCGTCAAATCCT
p53	XM_008767773.1	CCTTAAGATCCGTGGGCGT	GCTAGCAGTTTGGGCTTTCC
p16	NM_031550.1	TACCCCGATACAGGTGATGA	TACCGCAAATACCGCACGA

Supplementary Table S2. Primary and secondary antibodies used in this study (WB: Western blotting; ICC: Immunocytochemistry)

Antibodies	Purchase information	Host	Application and dilution
Anti-β-actin antibody	Proteintech	Mouse	1:1000 for WB
Anti-Aggrecan antibody	Norvus	Mouse	1:200 for ICC
Anti-Collagen II antibody	Abcam	Rabbit	1:200 for ICC
Anti-p53 antibody	Proteintech	Rabbit	1:1000 for WB
Anti-p16 antibody	Norvus	Mouse	1:1000 for WB
Anti-Akt antibody	CST	Mouse	1:1000 for WB
Anti-p-Akt antibody	CST	Rabbit	1:1000 for WB
Goat anti-mouse IgG	ZSGB-Bio, China	Goat	1:2000 for WB and 1:200 for ICC
Goat anti-rabbit IgG	ZSGB-Bio, China	Goat	1:2000 for WB and 1:200 for ICC

Supplementary Figure S1. Effects of PI3K/Akt pathway inhibition on cell viability and matrix biosynthesis of rat nucleus pulposus (NP) cells treated with TNF-α on day 1 and day 3. A: When the inhibitor LY294002 (10 μM) was added into the culture medium of NP cells in the 10 ng/mL TNF-α group, the activation of the PI3K/Akt

pathway was significantly inhibited. B: There was no significant difference in cell viability before and after inhibiting the activation of the PI3K/Akt pathway in NP cells treated with 10 ng/mL TNF-α. C-E: Inhibitor LY294002 up-regulated gene expression of matrix molecules aggrecan and collagen II (C), increased glycosaminoglycan (GAG) content (D) and increased matrix deposition in rat NP cells on day 1 and day 3 (E). The data are expressed as the mean±SD (n=3). *: Indicates a significant difference ($p<0.05$) between the 10 ng/mL TNF-α group and the 10 ng/mL TNF-α+LY294002 group.

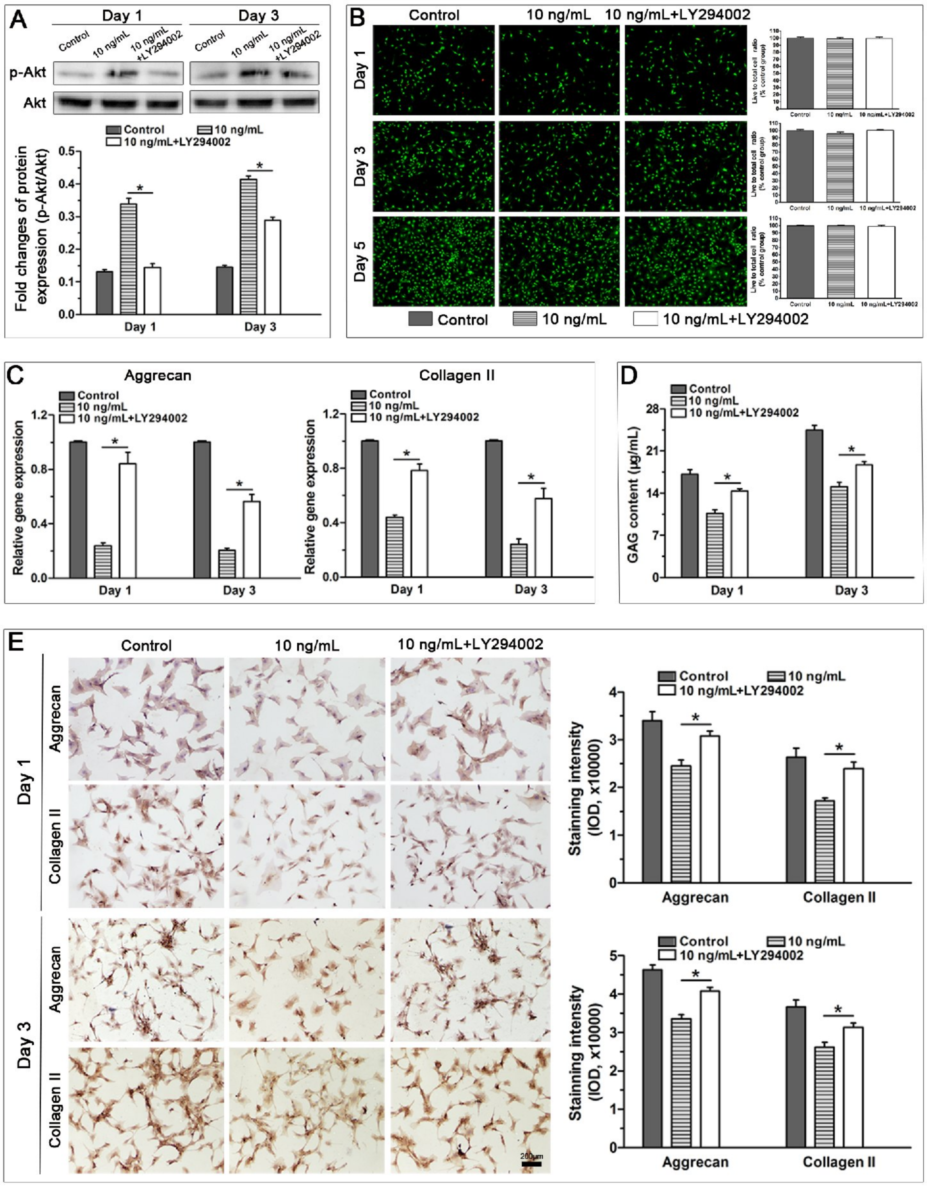

Supplement: Supplementary Figures and Tables [file srep42938-s1.pdf]
